# Bacterial Lipopolysaccharide Destabilizes Influenza Viruses

**DOI:** 10.1128/mSphere.00267-17

**Published:** 2017-10-11

**Authors:** Christopher Bandoro, Jonathan A. Runstadler

**Affiliations:** a Microbiology Graduate Program, Massachusetts Institute of Technology, Cambridge, Massachusetts, USA; b Department of Biological Engineering, Massachusetts Institute of Technology, Cambridge, Massachusetts, USA; c Division of Comparative Medicine, Massachusetts Institute of Technology, Cambridge, Massachusetts, USA; Emory University School of Medicine

**Keywords:** bacteria, environmental microbiology, influenza, lipopolysaccharide, microbiome, virology

## Abstract

Depending on the specific viral pathogen, commensal bacteria can promote or reduce the severity of viral infection and disease progression in their hosts. Influenza A virus (IAV) has a broad host range, comprises many subtypes, and utilizes different routes of transmission, including the fecal-oral route in wild birds. It has been previously demonstrated that commensal bacteria can interact with the host’s immune system to protect against IAV pathogenesis. However, it is unclear whether bacteria and their products may be interacting directly with IAV to impact virion stability. Herein we show that gastrointestinal (GI) tract bacterial isolates in an *in vitro* system significantly reduce the stability of IAV. Moreover, bacterial lipopolysaccharide (LPS), found on the exterior surfaces of bacteria, was sufficient to significantly decrease the stability of both human and avian viral strains in a temperature-dependent manner, including at the relevant temperatures of their respective hosts and the external aquatic habitat. The subtype and host origin of the viruses were shown to affect the extent to which IAV was susceptible to LPS. Furthermore, using a receptor binding assay and transmission electron microscopy, we observed that LPS binds to and alters the morphology of influenza virions, suggesting that direct interaction with the viral surface contributes to the observed antiviral effect of LPS on influenza.

**IMPORTANCE** Influenza A virus (IAV), transmitted primarily via the fecal-oral route in wild birds, encounters high concentrations of bacteria and their products. Understanding the extent to which bacteria affect the infectivity of IAV will lead to a broader understanding of viral ecology in reservoir hosts and may lead to insights for the development of therapeutics in respiratory infection. Herein we show that bacteria and lipopolysaccharide (LPS) interact with and destabilize influenza virions. Moreover, we show that LPS reduces the long-term persistence and freeze-thaw stability of IAV, which is important information for modeling the movement and emergence of novel strains from animal hosts. Our results, demonstrating that the subtype and host origin of a virus also influence its susceptibility to LPS, raise key questions about the fitness of viruses in reservoir hosts, their potential to transmit to humans, and the importance of bacterial-viral interactions in viral ecology.

## Abstract (precis)

Influenza A virus (IAV), transmitted primarily via the fecal-oral route in wild birds, encounters high concentrations of bacteria and their products. Understanding the extent to which bacteria affect the infectivity of IAV will lead to a broader understanding of viral ecology in reservoir hosts and may lead to insights for the development of therapeutics in respiratory infection. Herein we show that bacteria and lipopolysaccharide (LPS) interact with and destabilize influenza virions. Moreover, we show that LPS reduces the long-term persistence and freeze-thaw stability of IAV, which is important information for modeling the movement and emergence of novel strains from animal hosts. Our results, demonstrating that the subtype and host origin of a virus also influence its susceptibility to LPS, raise key questions about the fitness of viruses in reservoir hosts, their potential to transmit to humans, and the importance of bacterial-viral interactions in viral ecology.

## INTRODUCTION

Influenza A virus (IAV) is a global threat, infecting 5 to 10% of adults and 20 to 30% of children globally every year (1), and infections in humans and highly pathogenic IAV outbreaks in livestock substantially burden the economy (2, 3). All past pandemics of IAV in humans and outbreaks in livestock have origins in viruses that previously circulated among wild aquatic birds, which are the natural reservoir for the virus (4). In wild birds, the virus transmits primarily via the fecal-oral route (5). As a result, IAV encounters multiple environments, including the following environments: (i) the gastrointestinal (GI) tract inside the host; (ii) the feces in which the virus is shed; and (iii) the external aquatic habitats that the virus contaminates, facilitating transmission to new hosts. In these environments, virions encounter bacteria in abundances as low as 10^6^ bacteria/ml of freshwater (6) and upwards of 10^11^ bacteria/g inside the GI tract and feces (7, 8).

Studies examining the transkingdom interactions of commensal bacteria with human-pathogenic viruses demonstrate that bacteria can modify the severity of viral infection and disease progression in their hosts (9). Commensal bacteria promote the replication of fecally-orally transmitted picornaviruses, reoviruses, retroviruses, and noroviruses (10–13). In these cases, bacteria promote infection by interacting indirectly with the immune system and/or directly with the virions.

**FIG 1:**
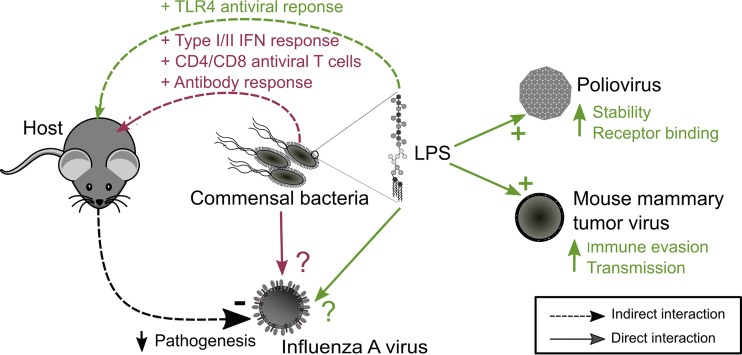
Summary of the indirect and direct interactions of bacteria and lipopolysaccharide on pathogenic viruses. A schematic representation summarizing the indirect and direct effects of bacteria and lipopolysaccharide (LPS) on influenza A virus (IAV) and other enteric viral pathogens is shown. Bacteria and LPS have been shown to indirectly limit the pathogenicity of IAV in mice through the different stimulating components of the immune system. Bacterial LPS has been demonstrated to interact directly with poliovirus and mouse mammary tumor virus, resulting in increased stability and transmission, respectively. The direct interactions and effects of bacteria and LPS with influenza virions are unclear. IFN, interferon.

Commensal bacteria and their products can indirectly protect against IAV infection by interacting with the host’s immune system (Fig. 1). In the absence of commensal bacteria, mice suffered from impaired type I/II interferon responses, CD4/CD8 T cell responses, and antibody production to IAV infection (14, 15). Moreover, mice pretreated with bacterial lipopolysaccharide (LPS), a product present on the exterior surface and shed by all Gram-negative bacteria, triggered a Toll-like receptor 4 (TLR4)-mediated antiviral response to protect the hosts from lethal infection with IAV (16, 17). In contrast, LPS was found to bind directly to the capsid protein of poliovirus, increasing cell attachment and the ability of the virions to remain infectious at elevated temperatures (18). Additionally, LPS binding to mouse mammary tumor virus (MMTV) resulted in increased immune evasion and transmission of the virus (13, 19). In the case of influenza, it is unclear whether commensal bacteria and LPS are interacting directly with IAV in addition to their indirect effects on the immune system.

Modeling studies have highlighted the importance of the indirect transmission route of IAV through contaminated aquatic habitats in establishing epidemics in birds and maintaining a reservoir of the virus (20–22). Abiotic factors, including temperature, pH, and salinity, that affect the long-term persistence of IAV in water are well established (23), but it is unclear how biotic factors, including bacteria and their products, affect virus stability (24). Keeler et al. observed that the long-term persistence of IAV was greater in filtered freshwater than in unfiltered water (25), suggesting that removing bacteria from water increases the stability of IAV.

In the present study, we report that GI tract commensal bacteria reduce the stability of IAV and that in contrast to results with other enteric viruses, bacterial LPS directly reduces the stability and long-term aquatic persistence of both avian and human influenza viruses. We also test the influence of subtype and host origin on the stability of IAV in LPS and show that LPS binds virions and affects their morphology. We propose that bacterial LPS has the ability to limit the transmission and infectivity of IAV directly, by interacting with virions, in addition to the previously described indirect effects through the immune system.

## RESULTS AND DISCUSSION

### Bacteria reduce the stability of IAV.

**FIG 2:**
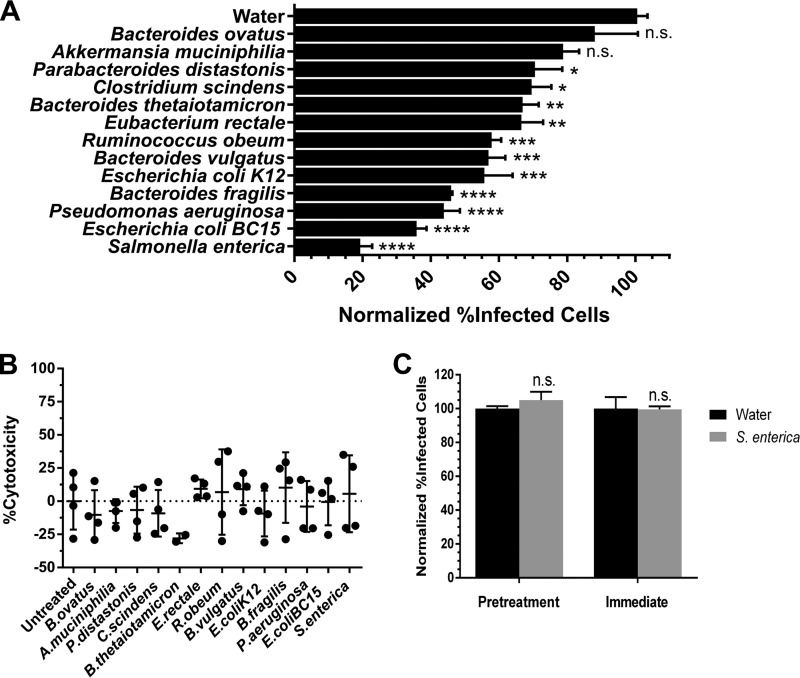
Commensal bacteria reduce the stability of influenza A virus. (A) Stability of a human H1N1 WSN-GFP virus after incubation for 1 h at 48°C with heat-killed gastrointestinal tract bacterial isolates normalized to a water control (*n* = 4). (B) A WST-1 assay was performed to assess the cytotoxicity of the heat-killed bacteria to MDCK-SIAT1-CMV-PB1 cells in the absence of virus after incubation overnight together (*n* = 2 to 4). (C) To test whether the reduction in stability observed was due to the bacteria affecting susceptibility of the host cells to infection, MDCK-SIAT1-CMV-PB1 cells were pretreated with diluted heat-killed *S. enterica* or water for 2 h at 37°C prior to infection or were infected immediately with a mixture of virus and 1 mg/ml of *S. enterica* cells or water (*n* = 3). Data are represented either as the data for individual replicates or the means plus standard errors of the means (SEM) (error bars). To assess statistical significance, a Dunnett’s multiple-comparison test or a Student’s *t* test was performed. Values that were significantly different are indicated by asterisks as follows: ****, *P* < 0.0001; ***, *P* < 0.001; **, *P* < 0.01; *, *P* <0.05. Values that were not significantly different (n.s.) are indicated.

We first tested whether GI tract-derived bacteria can affect the stability of IAV. Although the specific composition of the avian microbiome is quite distinct from that of mammals, more broadly, there are similarities at the level of individual phyla (26). Therefore, to broadly study the effects of bacteria on IAV stability, we selected a panel of 13 bacteria derived from the GI microbiome (Table 1) that are representative of the phyla present in human and avian microbiomes at the sites of IAV infection. For the lung microbiome in humans and the cloacal microbiome in mallards, *Firmicutes*, *Proteobacteria*, and *Bacteroidetes* were among the top three most commonly observed phyla in healthy individuals (27, 28). The bacteria selected in this study span the *Firmicutes*, *Bacteroidetes*, *Proteobacteria*, and *Verrumicrobia* phyla. A human WSN H1N1-GFP virus (see Materials and Methods) was incubated individually for 1 h at 48°C with these bacterial isolates, which were heat killed and standardized by protein content prior to incubation, or water control. We used infectivity as a measure of IAV stability which was assessed by measuring the percentage of infected cells (those expressing green fluorescent protein [GFP]) by flow cytometry after an overnight infection. Eleven of the thirteen bacterial strains significantly decreased the stability of the virus after incubation compared to that of the water control (Fig. 2A). Our results indicate the reduction in infectivity was not due to the diluted bacterial products being cytotoxic (Fig. 2B), nor was the decrease in infectivity due to the presence of bacteria or their products limiting the susceptibility of host cells to infection in the case of *Salmonella enterica* (Fig. 2C). This suggests that the observed reduction in stability at 48°C may be due to the virus interacting directly with the bacterial cells and/or their products.

**TABLE 1:** Bacterial strains used in this study

Bacterial species	Gram stain^a^	Phylum	Growth	Origin	Strain or isolate
*Akkermansia muciniphilia*	−	*Verrucomicrobia*	Anaerobic	Human	BAA 835
*Bacteroides fragilis*	−	*Bacteroidetes*	Anaerobic	Human	NCTC 9343
*Bacteroides ovatus*	−	*Bacteroidetes*	Anaerobic	Human	NCTC 11153
*Bacteroides thetaiotaomicron*	−	*Bacteroidetes*	Anaerobic	Human	VPI 5482
*Bacteroides vulgatus*	−	*Bacteroidetes*	Anaerobic	Mouse	ATCC 8482
*Clostridium scindens*	+	*Firmicutes*	Anaerobic	Human	VPI 13733
*Escherichia coli* BC 15	−	*Proteobacteria*	Aerobic	Mouse	BC 15
*Escherichia coli* K-12	−	*Proteobacteria*	Aerobic	Human	MG1655
*Eubacterium rectale*	+	*Firmicutes*	Anaerobic	Human	VPI 0990
*Parabacteroides distastonis*	−	*Bacteroidetes*	Anaerobic	Human	ATCC 8503
*Pseudomonas aeruginosa*	−	*Proteobacteria*	Aerobic	Human	PAO1
*Ruminococcus obeum*	+	*Firmicutes*	Anaerobic	Human	VPI B3-21
*Salmonella enterica* serotype Typhimurium	−	*Proteobacteria*	Aerobic	Human	LT2 ATCC 19585

a Symbols: –, negative; +, positive.

We observed that there were various degrees to which the individual bacterial isolates reduced IAV stability. Differences in IAV stability across the bacterial isolates could be explained by differences in the presence or quantity of various bacterial products that may be interacting with IAV. These bacterial products include but are certainly not limited to proteases, lipases, or polysaccharides that are cell wall associated, extracellular, or cytoplasmic in nature. Our method of heat inactivating the bacteria prior to incubation with IAV may be denaturing proteins and enzymes that would otherwise interact with the virus or that may be damaging the cell wall of the bacteria such that the virus is exposed to cytoplasmic bacterial products that would not happen normally. For the purposes of this study, we decided to focus on LPS, a polysaccharide that is produced universally by all Gram-negative bacteria. LPS is a heat-stable cell wall component (29) and should be unaffected by heat inactivation.

One can surmise that the specific composition of the microbial community and their interaction with IAV inside the host could dramatically modulate the infectivity of the virus. A recent study found that the cloacal microbiome of wild mallards infected with IAV differed from those who were IAV negative, specifically in the *Firmicutes*, *Proteobacteria*, and *Bacteroidetes* phyla (28). It is challenging to determine whether these differences in the microbiome preceded or were the result of IAV infection. The next steps should include testing the effects of bacterial communities of different complexity and composition derived from different IAV hosts on viral stability.

### Bacterial LPS interacts directly with IAV and reduces viral stability.

**FIG 3:**
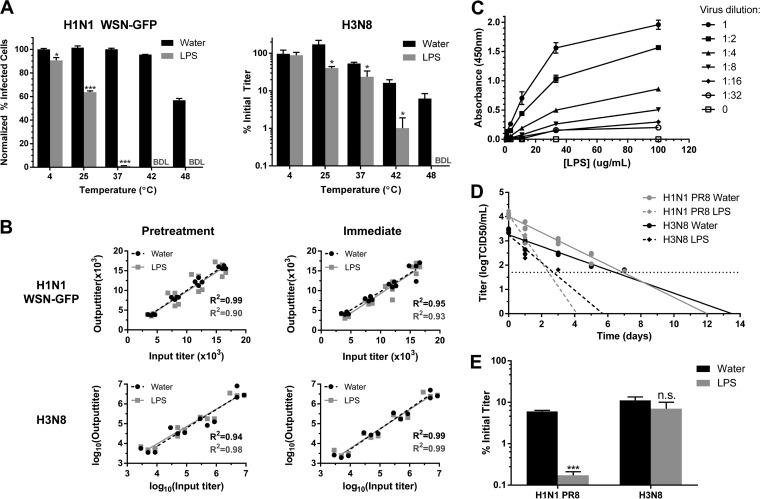
Bacterial lipopolysaccharide binds to and reduces the stability of influenza A virus. (A) Stability of a human H1N1 WSN-GFP virus and an avian H3N8 virus after incubation for 1 h at various temperatures with lipopolysaccharide (LPS) (*n* = 3). (B) Output titers of H1N1 WSN-GFP and H3N8, across a range of input titers, from tissue culture cells either (i) pretreated with LPS or water control and then infected or (ii) infected immediately after mixing the viruses with LPS or water (*n* = 4). BDL, below detectable limit. (C) Binding of biotinylated LPS to H1N1 PR8 after incubation for 1 h at 37°C (*n* = 2). (D) Long-term persistence of a human H1N1 PR8 and avian H3N8 in water (*n* = 13 or 12), or water containing diluted LPS (*n* = 6 or 7) at 25°C. The horizontal dotted line represents the limit of detection. (E) Aquatic freeze-thaw stability of a human H1N1 PR8 and avian H3N8 in LPS or water control (*n* = 4). Data are represented either as the data for individual replicates or as the means plus SEM. Statistical significance was assessed using a Student’s *t* test (A and E) or an F test on the linear regressions (B and D) and indicated as follows: ***, *P* < 0.001; *, <0.05, n.s., nonsignificant.

Given that bacterial LPS is present in milligram/milliliter concentrations in the animal GI tract (30) and that it interacts directly with poliovirus (10, 18) and mouse mammary tumor virus (MMTV) (13, 19), we hypothesized that LPS may be interacting with IAV and affecting its stability. Incubating a human WSN H1N1-GFP and an avian H3N8 subtype influenza virus with 1 mg/ml of *Escherichia coli* O111:B4 LPS for 1 h significantly reduced the stability of both viruses in a temperature-dependent manner (Fig. 3A). At these elevated temperatures, the conformation of viral proteins or the envelope may be changing and exposing domains that facilitate the interaction of LPS with the virion. Furthermore, with temperature held constant (37°C), LPS reduced the stability of IAV in a concentration-dependent manner (see Fig. S1 in the supplemental material). Although the estimated concentration of LPS in an animal GI tract is in the milligram/milliliter range (30), further studies comparing the concentration of LPS at the epithelial sites of infection for mammalian and avian hosts are necessary to ascertain the true physiological relevance of these results.

10.1128/mSphere.00267-17.1 FIG S1  LPS reduces the stability of H1N1 WSN-GFP in a concentration-dependent manner. A total of 1.0 × 10^6^ infectious units (IU)/ml of a human H1N1 WSN-GFP virus were added to increasing concentrations of *E. coli* LPS and incubated for 1 h at 37°C. Stability was evaluated for H1N1 WSN-GFP by flow cytometry following incubation to determine the percentage of cells expressing GFP, a measure of infectivity, which was then normalized to the values for samples not treated with LPS (0 µg/ml). Data are represented as means ± SEM (*n* = 4). Download FIG S1, TIF file, 0.1 MB. Copyright © 2017 Bandoro and Runstadler. 2017 Bandoro and Runstadler This content is distributed under the terms of the Creative Commons Attribution 4.0 International license.

We used our *in vitro* system to determine whether the observed reductions in infectivity in the stability assays were due to LPS interacting with the virus directly or through the indirect response of infected cells. Cells were either pretreated with LPS prior to infection or not pretreated with LPS and then were treated concurrently with a mixture of virus and LPS. Across a range of virus titers tested, the addition of LPS did not reduce the expected titer in our system for both scenarios (Fig. 3B), suggesting that the reduction in infectivity observed was due to LPS interacting directly with the virus, rather than the tissue culture cells mounting an antiviral response. This was further supported by the observation that detoxified LPS, a modified version of LPS lacking the immunogenic lipid A portion of the molecule, also reduced viral stability (Fig. S2). We also observed that biotinylated LPS binds to H1N1 PR8 virions in a solid-phase receptor binding assay (Fig. 3C). With 2.0 × 10^7^ virions, the dissociation constant (*K*_*d*_) was 24 µg/ml of LPS (*R*^2^ = 0.99). Since the hemagglutinin (HA) glycoprotein of IAV binds to sialylated receptors on host cells, we postulated that LPS binding is mediated through HA. However, pretreating the virus with antibodies against HA or its sialic acid receptors failed to block binding to LPS (Fig. S3). LPS could be interacting with virions in an HA-independent manner, either through the viral neuraminidase or potentially through host proteins derived from the host cell membrane as with MMTV (19).

10.1128/mSphere.00267-17.2 FIG S2  Nonimmunogenic LPS also reduces the stability of influenza A viruses. For a control, to test whether nonimmunogenic LPS reduced influenza virus stability, 1.0 × 10^6^ IU/ml of H1N1 WSN-GFP or 5.0 × 10^6^ TCID_50_/ml H3N8 virus were added to either water or 1 mg/ml of *E. coli* detoxified LPS and incubated for 1 h at 37°C and 42°C, respectively (*n* = 3 or 4). The respective stabilities for the viruses are displayed. Below detectable limit (BDL) indicates that the samples fell below the detectable limit of 0.75% normalized infected cells for H1N1 WSN-GFP or 0.025% initial titer for H3N8. Data are represented as mean ± SEM in all panels. Statistical significance was assessed using a Student’s *t* test to compare the values for virus treated with LPS and water (control). ***, *P* < 0.001. Download FIG S2, TIF file, 0.2 MB. Copyright © 2017 Bandoro and Runstadler. 2017 Bandoro and Runstadler This content is distributed under the terms of the Creative Commons Attribution 4.0 International license.

10.1128/mSphere.00267-17.3 FIG S3  Antihemagglutinin antibodies and receptor binding pocket inhibitors do not block lipopolysaccharide binding. To evaluate whether the receptor-binding site of hemagglutinin (HA) mediated lipopolysaccharide (LPS) binding, influenza H1N1 PR8 virions were pretreated for 1 h at room temperature (RT) with dilutions of antibodies against HA head and H1N1 (starting concentrations of 1 mg/ml and 5 mg/ml), the sialic acid receptors of HA, α2,6- and α2,3-sialyllactose (starting concentration of 1 mM), or PBS control prior to incubation with biotinylated LPS for 1 h at 37°C. LPS binding was detected using HRP-streptavidin, TMB substrate, and 0.2 M sulfuric acid. Absorbance was measured at 450 nm and normalized to the PBS control (*n* = 2 to 4). Data are represented as mean ± SEM. Download FIG S3, TIF file, 0.4 MB. Copyright © 2017 Bandoro and Runstadler. 2017 Bandoro and Runstadler This content is distributed under the terms of the Creative Commons Attribution 4.0 International license.

Aquatic habitats serve as long-term reservoirs for avian IAVs and are important to sustain transmission of the viruses in wild birds (20, 21). In our study, avian H3N8 and human H1N1 PR8 viruses persisted significantly longer in the water control than in water containing 100 µg/ml of LPS at 25°C (*P* = 0.002 and <0.001, respectively) (Fig. 3D). Using the slopes of the regression analyses, for each treatment we calculated the log_10_ reduction time (Rt), defined as the length of time for the viral titer to decrease by 90% (31). LPS reduced the Rt of the avian virus in water compared to the Rt of virus in the water control by 58% (Rt of virus in water [Rt_water_] = 4.2 days; Rt of virus in water containing LPS [Rt_LPS_] = 1.8 days), and to a greater extent, by 66%, for the human H1N1 (Rt_water_ = 3.0 days; Rt_LPS_ = 1.0 day). Although this experiment was conducted at 25°C, infectious IAV has previously been found to persist much longer, on the order of hundreds of days, at colder temperatures of 4 to 10°C (31–33). In light of the natural history of IAV, in future studies, it would be interesting to evaluate the degree to which bacterial LPS could influence the persistence of IAV at these colder temperatures over much longer periods of time.

Viruses circulating in the aquatic environment must also be able to withstand the natural freeze-thaw cycles that rapidly inactivate the virions (34). We found that LPS significantly reduces the freeze-thaw stability of the human H1N1 PR8 virus by 34-fold compared to the stability of virus in the water control (*P* < 0.001), whereas the avian H3N8 virus was not significantly affected (Fig. 3E). Bacterial products such as LPS may be reducing persistence and stability of IAV in the environment, which has significant implications for modeling the emergence of novel strains from animal reservoir hosts.

### Subtype and host origin affect LPS susceptibility.

**FIG 4:**
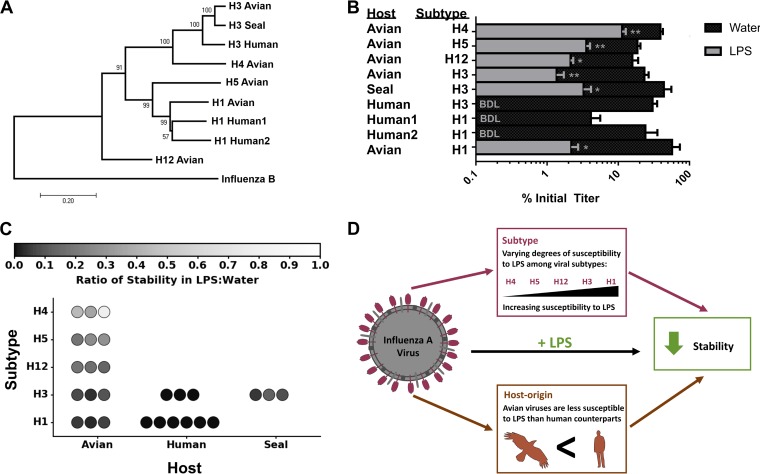
Host origin and subtype affect the susceptibility of influenza A virus to lipopolysaccharide. (A) Phylogenetic tree constructed using the hemagglutinin (HA) gene of nine influenza A viruses with influenza B virus included as an outgroup. Bootstrap percentages for the internal nodes are displayed. The bar shows the evolutionary distance (number of base substitutions per site). (B) Stabilities of these viruses after incubation for 1 h at 48°C in lipopolysaccharide (LPS) or water control (*n* = 3). Data are represented as means plus SEM. Statistical significance was assessed using a Student’s *t* test to compare the values for virus treated with LPS and water control. **, *P*  < 0.01;  *, *P* < 0.05. BDL, below detectable limit of 0.025% initial titer. (C) Ratio of stability in LPS and water of the viruses across hosts and subtypes. The value for each replicate is represented as a circle. (D) Schematic representation summarizing the results.

Our long-term aquatic viral persistence and freeze-thaw stability results suggested that the avian isolate may be more resistant to LPS than the human virus tested. In addition, it has been reported that the microbiome content of IAV-infected mallards significantly differed between HA types of the virus (28). To examine the extent to which susceptibility to LPS differs between viruses, we compared the stabilities across a panel of viruses (Fig. 4A) that represented (i) phylogenetically distinct HA subtypes H1, H3, H4, H5, and H12 and (ii) different host origins (avian, human, seal). All of the strains tested had significantly reduced stabilities in water containing *E. coli* LPS compared to the water control after incubation at 48°C for 1 h (Fig. 4B), suggesting that LPS appears to have a universal effect of reducing the stability of influenza viruses.

Measurements of relative stability of each virus in water containing LPS compared to the relative stability of each virus in the water control (LPS/water stability) did not follow a normal distribution (*P* < 0.05 by the Shapiro-Wilks test) and were tested with a nonparametric Kruskal-Wallis H test. A significant difference was detected between subtypes (*P* < 0.001), as well as host origin (*P* < 0.001). The H4 subtype, followed by the H5 and H12 subtypes (all avian origin) showed the highest stability in LPS, compared to the H1 and H3 subtypes (Fig. 4C). We further analyzed the H1 and H3 subtypes, because for these two subtypes measurements of LPS/water stability were made for two or more hosts. For subtype H1, virus originating from avian hosts was significantly more stable in water containing LPS compared to human viruses (df = 1; *P* = 0.006). For subtype H3, avian and seal viruses were more stable in LPS compared to human viruses, though not at a statistically significant level (df = 2; *P* = 0.055). Different strains of the viral subtypes in our panel were not tested. It has previously been reported that there are strain-related differences in the persistence of IAV in water (25); therefore, we cannot rule out the possibility that the differences in LPS susceptibility may be due to strain effects rather than subtype effects.

It is important to note that the viruses used in this panel were propagated in different laboratory hosts, either embryonated chicken eggs (ECEs) or MDCK cells (Table 2). It has been suggested that viruses passaged in different host cells may differ in their lipid content which could affect viral stability in water (35). We observed a significant difference between propagation hosts on LPS susceptibility (*P* = 0.02), but this may due to the fact that the majority of the avian and human viruses were grown in ECEs and MDCK cells, respectively. The one avian H3 virus propagated in MDCK cells displayed reduced susceptibility to LPS and the one human H3 virus propagated in ECEs displayed increased susceptibility, indicating that the propagation hosts are not entirely responsible for our observed results.

**TABLE 2:** Influenza A virus strains used in this study

HA subtype	Host	IAV subtype	Strain	Passage history^a^	Segment 4 (HA) GenBank accession no.
H1	Human 1^b^	H1N1	A/Puerto Rico/8/1934	P2 MDCK	LC120391
	Human 2^b^	H1N1	A/Netherlands/2629/2009	P1 ECE	CY065784
	Avian	H1N1	A/mallard/Interior Alaska/12ML00957/2012	P1 ECE	KY750593
H3	Avian	H3N8	A/mallard/Interior Alaska/10BM11415R0/2010	P3 MDCK	CY143893
	Seal	H3N8	A/harbor seal/New Hampshire/179629/2011	P2 MDCK	KJ467564
	Human	H3N2	A/Brisbane/10/2007	P1 MDCK	KM978061
H5	Avian	H5N2	A/mallard/Interior Alaska/12ML01123/2012	P1 ECE	KY750601
H4	Avian	H4N6	A/mallard/Interior Alaska/12ML00831/2012	P1 ECE	KY750585
H12	Avian	H12N5	A/mallard/Interior Alaska/12ML00678/2012	P1 ECE	KY750577

a P2, passage 2; MDCK, Madin-Darby canine kidney cells; ECE, embryonated chicken eggs.

b 1 and 2 are used here to differentiate the two human H1 viruses in this experiment.

Our data suggest that subtype and host origin of IAV influence the ability of the virus to withstand inactivation (Fig. 4D). Most notably, IAV strains derived from birds were more stable in water containing LPS than the strains derived from humans. The one exception was the mammalian seal H3 virus, which was also more resistant to LPS (Fig. 4B). However, upon further inspection, this virus descended from viruses circulating in North American waterfowl (36) and has retained its avian-like receptor specificity (37). Therefore, it is interesting that this seal H3 also appears to retain avian-like resistance to LPS. Given that avian IAV transmits primarily via the fecal-oral route (5) compared to the respiratory transmission of human IAV (38) and that the bacterial load is higher in the GI tract than airways (26, 39), avian strains may be adapted to the high concentrations of LPS that they encounter inside the host and in feces. Although the focus of this study was primarily with LPS and the interaction with virus, in the future it would be valuable to compare the stability of a broader panel of viruses with bacteria to fully investigate this hypothesis.

### Incubation with LPS alters the morphology of influenza virions.

**FIG 5:**
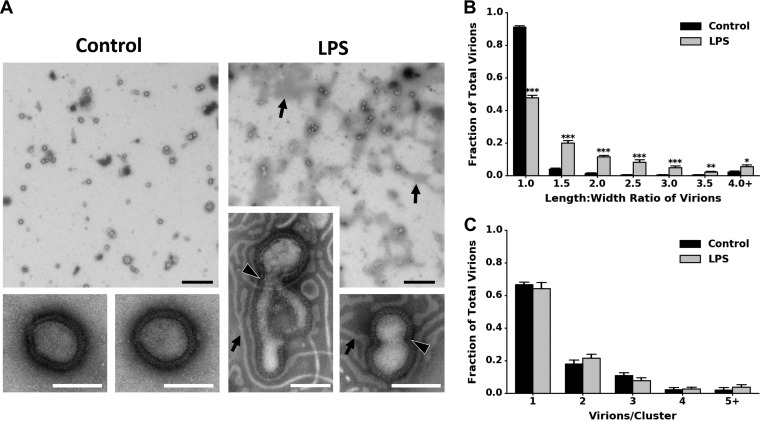
Bacterial lipopolysaccharide affects the integrity of the viral envelope but does not affect aggregation. (A) Transmission electron micrographs of H1N1 PR8 after incubation for 1 h at 37°C in either water (control) or lipopolysaccharide (LPS). Representative micrographs were taken at magnifications of ×4,800 and ×49,000. LPS ribbons (black arrows) and compromised virion membranes (black arrowheads outlined in white) are labeled. Bars, black, 1 µm; white, 100 nm. (B and C) Fractions of total virions in water control (*n* = 559) and LPS (*n* = 579) were binned by the length/width ratio of the virions to assess differences in viral envelope morphology (B) or by the number of virions per viral cluster to assess differences in virion aggregation (C). Fractions of virions were calculated based on the total number of virions in each micrograph. Data are represented as the mean plus SEM of 9 to 10 micrographs. Statistical significance was assessed by a Student’s *t* test and indicated by asterisks as follows: ***, *P*  < 0.001; **, *P* < 0.01; *, *P* < 0.05.

Because LPS binds directly to IAV and reduces the stability of the virus in a temperature-dependent manner, we hypothesized that LPS may be compromising the structural integrity of the virions’ envelopes. H1N1 PR8 virions incubated in water at 37°C for 1 h retained their spherical morphology by transmission electron microscopy (TEM), whereas virions incubated with LPS had envelopes that were more noticeably deformed (Fig. 5A). To quantify whether there was a change in virion morphology, we measured the length/width ratio of the virions (Fig. S4). LPS-treated virions had a significantly different length/width ratio distribution compared to control virions using a Kolmogorov-Smirnov (KS) test (*P* < 0.001) (Fig. 5B) but did not result in increased virion aggregation (Fig. 5C). Therefore, LPS causes an increase in the proportion of virions with envelope deformations, which may account for the decrease in stability observed.

10.1128/mSphere.00267-17.4 FIG S4  Example measurements and calculations of the length/width ratio of influenza virions. Virions were negatively stained and observed by transmission electron microscopy. The length (pink), across the longest axis of the virions, and its bisecting width (green) were measured for virions incubated in water (control) and LPS for 1 h at 37°C. The distributions for these length/width ratios are presented in Fig. 5B. Control virions that are round should have a ratio of ~1.0, and those with elongated morphologies should have a ratio of >1. Download FIG S4, TIF file, 0.8 MB. Copyright © 2017 Bandoro and Runstadler. 2017 Bandoro and Runstadler This content is distributed under the terms of the Creative Commons Attribution 4.0 International license.

How does LPS binding to the IAV result in the observed damage to the virions’ envelopes? We propose two possible mechanisms. First, LPS may be interacting directly with the lipid envelope of IAV, potentially constricting the envelope and resulting in the morphological changes observed. Second, although we have shown that LPS is not binding to the receptor binding site of HA, it may be interacting with other domains of HA. When IAV is exposed to low pH, conformational changes in HA reserved for fusion in the endosome follow, resulting in dramatic changes to virion morphology and even virions fusing together (40). Supposing LPS binding to HA at increased temperatures triggers similar conformation changes in HA, this may explain the deformed virions observed as well as the longer virions, which may represent fused virions.

At this moment, it is unclear which stage of IAV infection is being limited by LPS binding to virions directly and altering their morphology. Due to the alterations in the morphology of the viral envelope observed, we would hypothesize that virions bound with LPS have reduced binding or fusion to the host cell. However, it is possible that bound LPS may also be limiting downstream replication stages of IAV.

In conclusion, our study provides new evidence that LPS directly affects the stability of IAV by binding directly to and altering the morphology of influenza virions, suggesting that bacteria within hosts and in the external environment can limit the transmission of influenza virus. These findings are in contrast to those observed with poliovirus (10, 18), leading us to propose that interaction with LPS is highly dependent on virion structure. Our results are presented mainly in the context of IAV transmission within its natural avian reservoir; however, we found that human-derived viruses are more susceptible to bacterial products. Understanding the extent to which bacteria and their products interact with and affect the infectivity of IAV may suggest novel pathways for the development of novel therapeutics to prevent or treat respiratory infections. It is important to highlight that all three of the Gram-positive bacteria, which lack LPS, tested in this study also reduced the stability of IAV. Robinson et al. found that in addition to LPS, Gram-positive bacterial peptidoglycan enhanced the stability of poliovirus (18), suggesting that other bacterial polysaccharides may also be interacting with IAV.

Moreover, the overuse of antibiotics in poultry and swine, in addition to generating and disseminating antibiotic-resistant strains of bacteria (41), may be increasing their risk of IAV infection. Additional studies monitoring the effects of antibiotics and changes in the microbiome on the susceptibility of animals, including humans, to pathogenic viruses will help clarify the extent to which bacterium-virus interactions may be influencing the severity and epidemiology of viral infection.

## MATERIALS AND METHODS

### Bacterial strains, viruses, and cell lines.

Cultures of GI tract and fecal microbiome bacterial isolates from humans and mice (Table 1) were heat killed at 95°C for 10 min and standardized by their protein content using a bicinchoninic acid (BCA) assay (Thermo Fisher). Viruses used in this study are listed in Table 2. We also used a reverse-genetics-engineered H1N1 WSN PB1flank-eGFP (obtained from the laboratory of Jesse Bloom), referred to in this study as H1N1 WSN-GFP, in which the PB1 gene’s coding sequence was replaced by a gene encoding enhanced green fluorescent protein (eGFP) (42). Viruses were propagated in either Madin-Darby canine kidney (MDCK) cells (obtained from American Type Culture Collection), MDCK-SIAT1-CMV-PB1 cells (obtained from the laboratory of Jesse Bloom), or embryonated chicken eggs (ECEs) (obtained from Charles River Laboratories) for 72 h. The avian H3N8 and human H1N1 PR8 were further purified and concentrated through a 30% sucrose cushion during ultracentrifugation (43).

### Ethics statement.

Cultures of GI tract and fecal microbiome bacterial isolates were obtained from an existing collection of isolates from the laboratory of Timothy Lu. The sources of the bacterial isolates from humans were anonymized. Embryonated chicken eggs, obtained from a vendor (Charles River Laboratories), were infected on days 9 to 11 during development as reviewed and approved by the Committee on Animal Care at the Massachusetts Institute of Technology (protocol number E15-02-0218).

### Growth and infection media.

MDCK cells and MDCK-SIAT1-CMV-PB1 cells were grown in Dulbecco modified Eagle medium (DMEM) (HyClone) supplemented with 10% fetal bovine serum (FBS) (Seradigm), and 100 U/ml penicillin and 100 µg/ml streptomycin (Sigma) at 37°C with 5% CO_2_. H1N1 WSN-GFP infections were carried out in MDCK-SIAT1-CMV-PB1 cells in Opti-MEM (HyClone) supplemented with 0.01% FBS, 0.3% bovine serum albumin (BSA), 100 U/ml penicillin, 100 µg/ml streptomycin, and 100 µg/ml calcium chloride at 37°C with 5% CO_2_. Infections involving all the other viruses were carried out in DMEM supplemented with 0.2% BSA (Thermo Fisher), 25 mM HEPES (Corning), 100 U/ml penicillin, and 100 µg/ml streptomycin at 37°C with 5% CO_2_.

### Virus titers and infectivity.

The titers of H1N1 WSN-GFP virus were initially determined as described previously (42). Briefly, serial dilutions of the stock virus were allowed to infect 1.0 × 10^5^ MDCK-SIAT1-CMV-PB1 cells for 16 h at 37°C with 5% CO_2_. The percentages of infected cells were determined by flow cytometry (Accuri C6; Accuri Cytometers), using uninfected control cells as the baseline and setting 0.5% of the control cells as GFP positive (FlowJo). We then used the Poisson equation to calculate the number of infectious units (IU) per milliliter of the virus in initial stock. For the stability assays, viruses were added to cells as described above along with a standard curve of known quantities of virus in parallel to ensure that they were in the linear range of the assay. To compare samples across independent replicates, we normalized the percentages of infected cells to that of the water control. The titers of all other viruses were determined in MDCK cells by 50% tissue culture infective dose (TCID_50_) assays (44). Briefly, 3.0 × 10^4^ MDCK cells were seeded into 96-well plates (VWR) overnight, and then viruses were serially diluted across the plate, incubated for 2 h to allow attachment, washed, and then returned to incubate for 72 h at 37°C with 5% CO_2_. The presence or absence of cytopathic effect (CPE) was observed, and the TCID_50_/milliliter was calculated using the Reed-Muench method (45).

### Stability assays.

To test whether bacterial products may be affecting the stability of influenza virus, 1.0 × 10^6^ infectious units/ml of H1N1 WSN-GFP was mixed into water or the heat-killed bacterial isolates, standardized to 1 mg/ml protein in water, and incubated at 48°C for 1 h. Similarly, to test the effects of LPS on influenza virus stability, 1.0 × 10^6^ IU/ml of H1N1 WSN-GFP or 5.0 × 10^6^ TCID_50_/ml of the other viruses (Table 2) were spiked into either water alone or water containing 1 mg/ml of purified *E. coli* O111:B4 LPS (Sigma) at a range of temperatures for 1 h. Stability for H1N1 WSN-GFP was measured by determining the percentage of infected cells, normalized to that in the water control, by flow cytometry. For all other viruses, we calculated the percentage of the initial titer remaining after incubation (final titer/initial titer × 100%) by TCID_50_ assays as a measure of stability. For all stability trials, Student’s *t* tests were applied to determine whether there was significant difference between the virions in water alone and water containing LPS. To compare the stabilities of the panel of IAV strains tested, we calculated the ratio of their stability in water containing LPS and water alone (percent initial titer in water containing LPS/percent initial titer in water).

### Stability controls.

For a control to determine whether the change in infectivity was caused by bacteria or LPS affecting the susceptibility of the MDCK or MDCK-SIAT1-CMV-PB1 cells, we examined the output titers of a range of known input titers of avian H3N8 and human H1N1 WSN-GFP. The cells were pretreated for 2 h at 37°C (the length of time the cells were incubated with viruses plus LPS in the assay before being washed) with diluted heat-killed *S. enterica* or *E. coli* LPS prior to infection, or the cells were infected immediately with mixtures of the viruses and 1 mg/ml of *S. enterica* or LPS. An F test was performed on linear regressions of the samples pretreated with LPS and water or immediately infected with virus in water containing LPS and water alone to test whether the slopes of the lines were significantly different. A WST-1 assay (G-Biosciences) was used to assess the cytotoxicity of the bacteria to the cells without the presence of virus. For an additional control, virions were also incubated with 1 mg/ml of detoxified *E. coli* O111:B4 LPS (Sigma) which is missing the immunogenic lipid A portion.

### Aquatic environmental stability.

For the long-term persistence experiment, 1.0 × 10^4^ TCID_50_/ml of avian H3N8 or human H1N1 PR8 were added to water alone or water with 100 µg/ml of LPS and incubated at 25°C. Over 7 days, viruses were periodically removed and the virus titers were determined immediately to determine the quantity of infectious virions remaining. Data points were log_10_ transformed and then fit with a linear regression using GraphPad to calculate the log_10_ reduction time (Rt), which is the time required for infectivity to decrease by 90% (or 1 log_10_ TCID_50_/ml) (31, 46). An F test was performed to compare the slopes of the linear regressions.

Freeze-thaw stability was tested by adding 1.0 × 10^6^ TCID_50_/ml of human H1N1 PR8 or avian H3N8 virus to either water alone or water containing 1 mg/ml of *E. coli* LPS. Samples were frozen at −20°C for 3 to 5 days and then thawed at 25°C. Stability was assessed in a manner similar to that in the above stability experiments by measuring the titer prior to freezing and after thawing the viruses.

### Phylogenetic analysis.

A panel of viruses (Table 2) was selected based on diversity of hemagglutinin (HA) subtypes and host origin of the viruses. The nucleotide sequences of the viruses’ segment 4 genes, which encode HA, were used to construct a phylogenetic tree. We used MEGA7 (47) to compute the evolutionary distances via maximum composite likelihood method (48) and assessed the evolutionary history using the neighbor-joining method (49). Influenza B virus B/Durban/39/98 was included as an outgroup in the analysis.

### Binding.

To test whether LPS binds to virions, we used a modified version of a solid-phase influenza receptor binding assay described previously (4). Briefly, H1N1 PR8 virions, at various dilutions, were bound to fetuin A (Sigma)-coated wells overnight at 4°C. After the wells were washed, dilutions of biotinylated *E. coli* O111:B4 LPS (InvivoGen) were added and incubated at 37°C for 1 h. LPS binding was detected using horseradish peroxidase (HRP)-streptavidin (Thermo Fisher), 3,3′,5,5′-tetramethylbenzidine (TMB) substrate (Thermo Fisher), and 0.2 M sulfuric acid. Absorbance was measured at 450 nm. Binding *K*_*d*_ was calculated using a nonlinear regression with GraphPad software. To assess whether LPS binding was mediated by HA, virions were pretreated for 1 h at room temperature prior to LPS with dilutions of monoclonal antibodies against the globular HA head (anti-HA head; provided by Peter Palese’s lab), polyclonal antibodies against H1N1 (anti-H1N1; Thermo Fisher), or with α2,6- and α2,3-sialyllactose (Carbosynth).

### Transmission electron microscopy.

A total of 1.0 × 10^8^ TCID_50_/ml of H1N1 PR8 virions were mixed with 1 mg/ml of *E. coli* LPS or water control and then incubated at 37°C for 1 h. Carbon/Formvar film supported Cu grids were glow discharged and then placed on a drop of influenza virus mixtures and then stained with 1% (wt/vol) phosphotungstic acid. Samples were examined using a Tecnai G2 Spirit BioTWIN transmission electron microscope, and representative transmission electron microscopy (TEM) images were taken at magnifications of ×4,800 and ×49,000. To test whether LPS was affecting the shape of the virions, the length and the perpendicular bisecting width of the virions were measured using ImageJ at a magnification of ×4,800 across micrographs for each treatment. The ratio of length to width of each virion was calculated. An example can be seen in Fig. S4 in the supplemental material. To determine whether LPS was affecting the aggregation of virions, virions in different viral clusters (cluster sizes of 1, 2, 3, etc.) were enumerated as described above for each treatment. The fractions of virions in each length/width ratio bin or cluster were calculated and normalized based on the total number of virions in each micrograph.

### Statistical analyses.

Statistical analyses were performed by using GraphPad Prism 6 software, JMP Pro 12, and Python v2.7 with Numpy v1.12 and Scipy v0.18. Student’s *t* tests were performed to compare the means between two groups (parametric). To compare multiple groups, we assessed normality via a Shapiro-Wilks test and then computed a one-way analysis of variance (ANOVA) followed by a posthoc Dunnett’s multiple-comparison test (parametric) or Kruskal-Wallis H test (nonparametric). To compare distributions, we used the nonparametric Kolmogorov-Smirnov (KS) test to test the null hypothesis that the distributions were the same. Statistical significance threshold was assessed at *P* values of <0.05. See the figure legends or tables to find the statistical tests, exact values of *n*, what *n* represents, and the definitions of center, dispersion, and precision measurements.

### Accession number(s).

New sequence data for four avian hemagglutinin genes used in the phylogenetic analysis are available in GenBank: A/mallard/Interior Alaska/12ML00957(H1N1)/2012 (GenBank accession no. KY750593), A/mallard/Interior Alaska/12ML01123(H5N2)/2012 (GenBank accession no. KY750601), A/mallard/Interior Alaska/12ML00831(H4N6)/2012 (GenBank accession no. KY750585), and A/mallard/Interior Alaska/12ML00678(H12N5)/2012 (GenBank accession no. KY750577).
