# Supplementary figures and images for: Bacterial Lipopolysaccharide Destabilizes Influenza Viruses
Source: mSphere. 2017 Oct 11;2(5):e00267-17. doi: 10.1128/mSphere.00267-17 (PMC5636225; doi:10.1128/mSphere.00267-17)

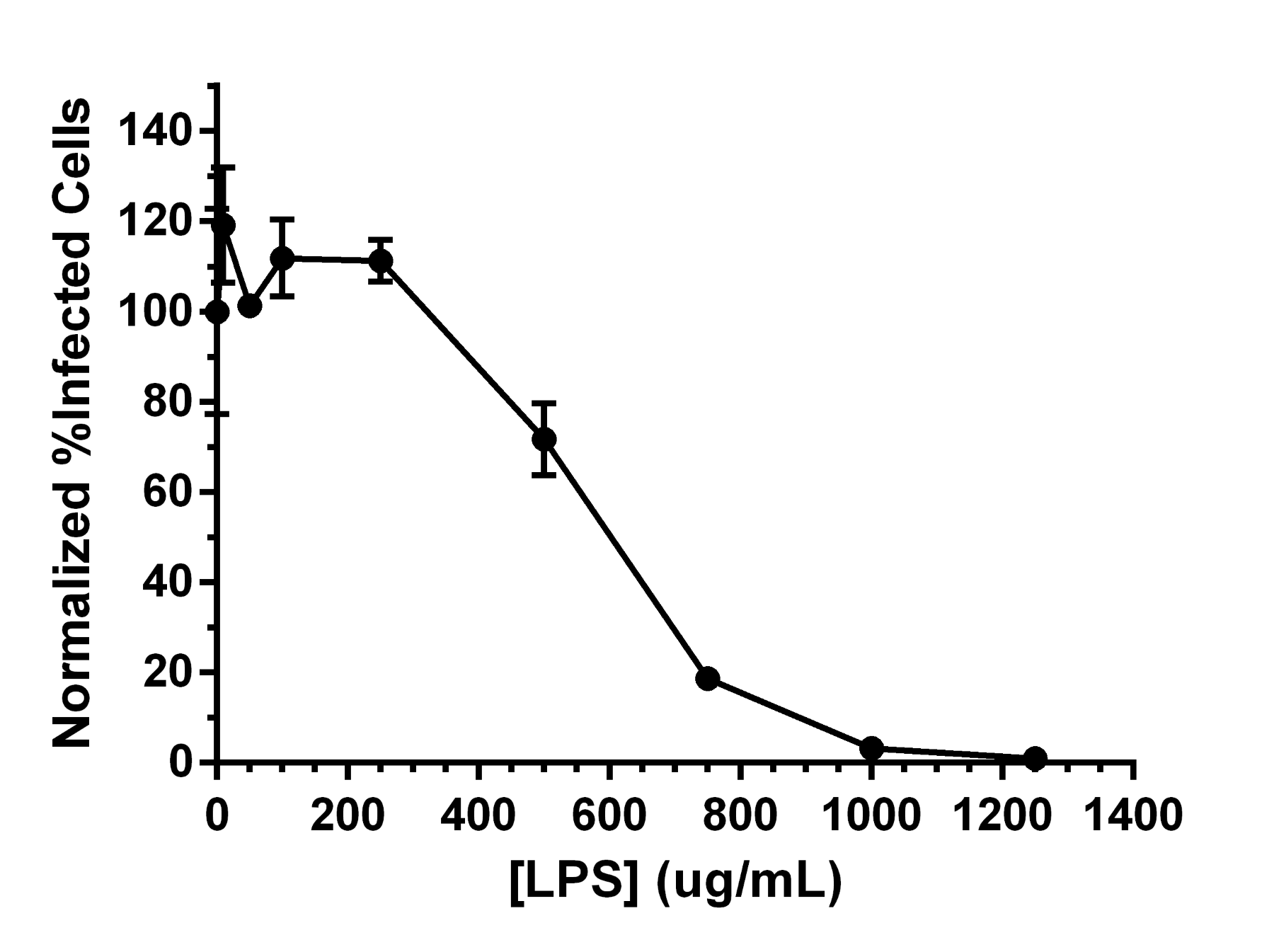

Supplement: FIG S1 [file sph005172379sf1.tif]

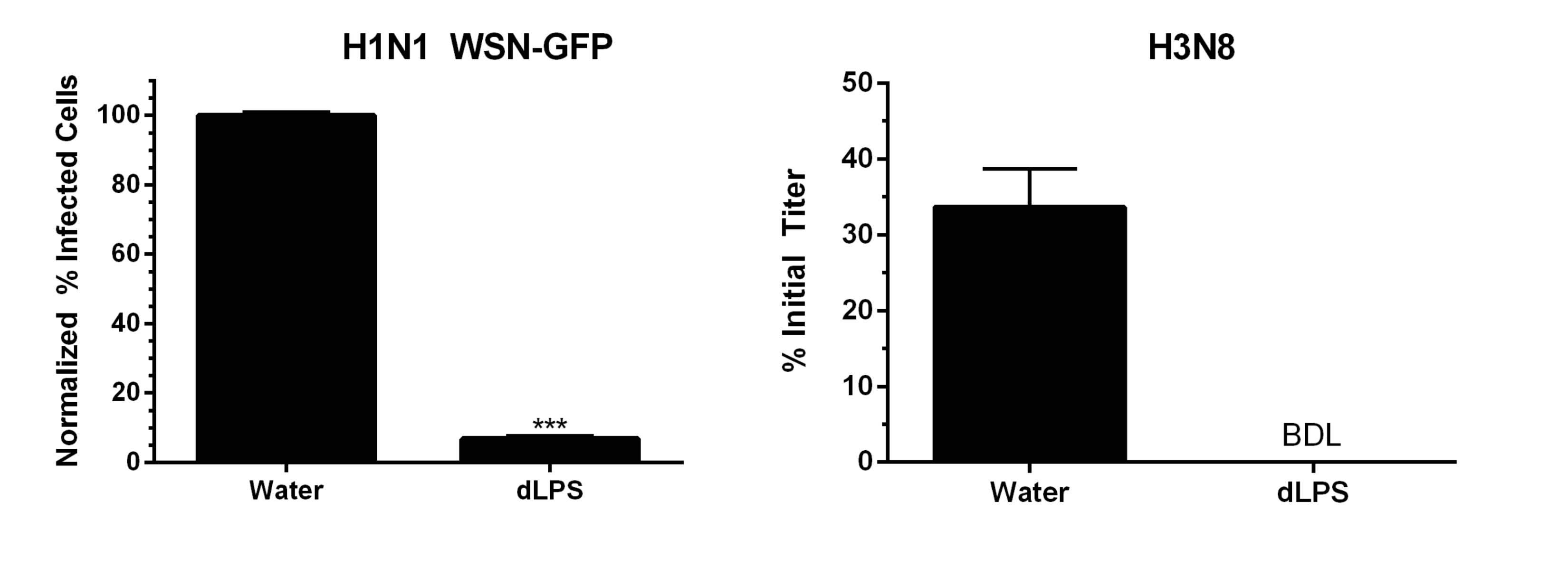

Supplement: FIG S2 [file sph005172379sf2.tif]

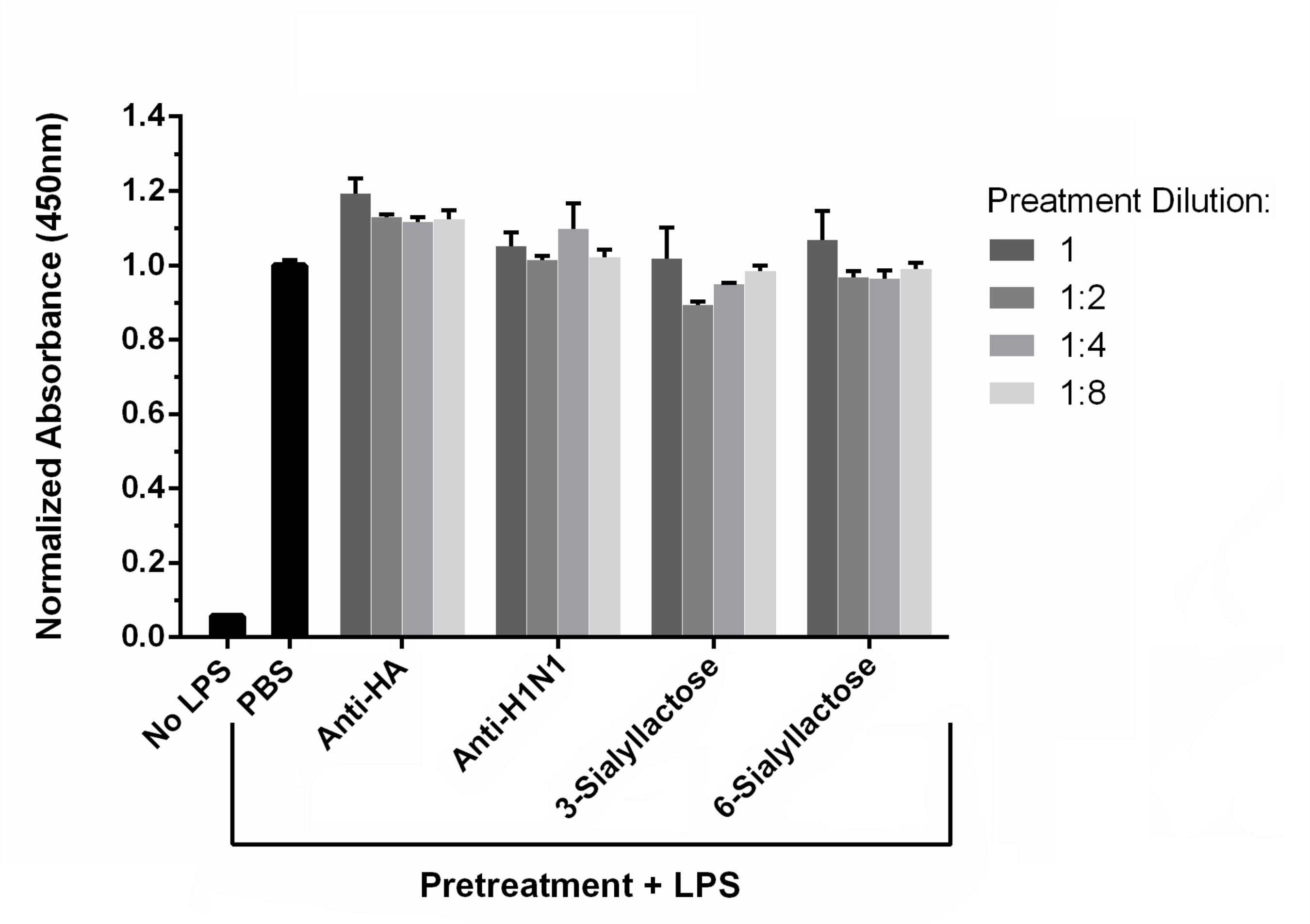

Supplement: FIG S3 [file sph005172379sf3.tif]

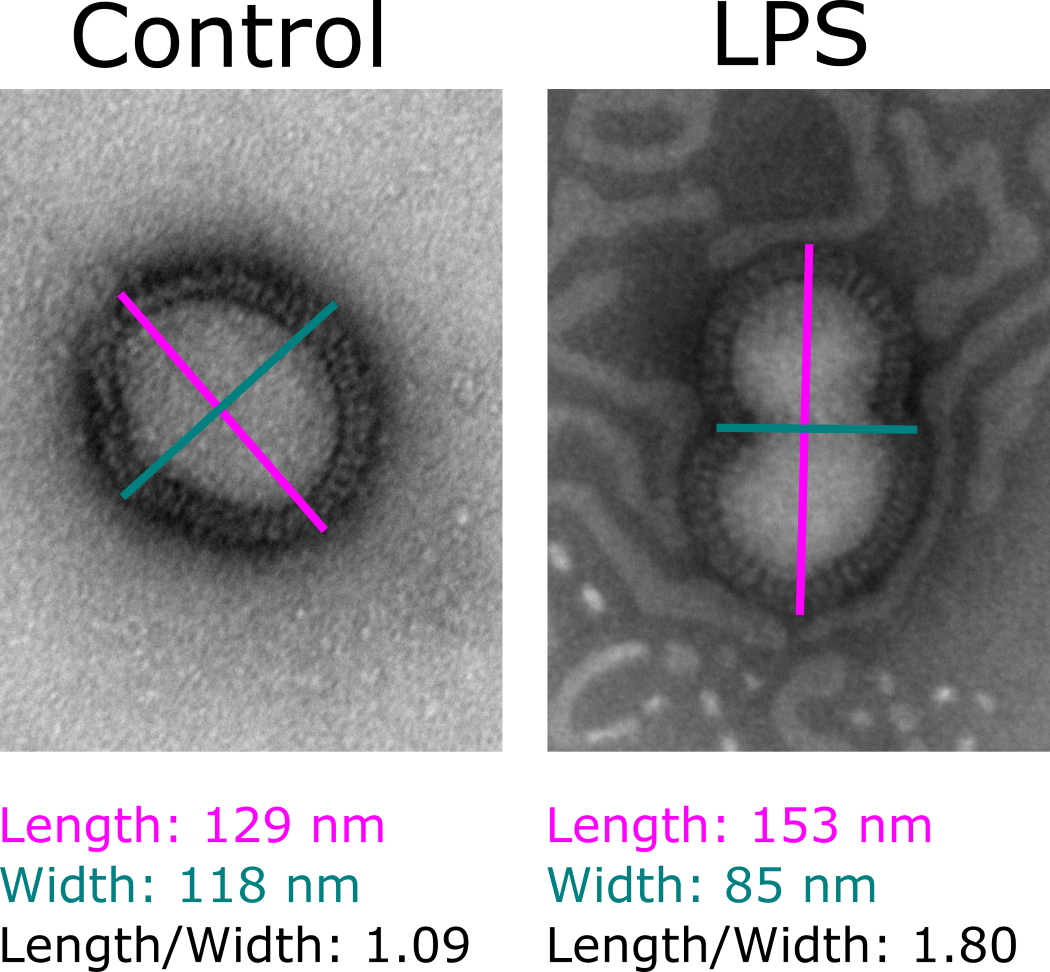

Supplement: FIG S4 [file sph005172379sf4.tif]
